# Reciprocal Regulation of Peroxisome Biogenesis and Myogenic Factors Is Critical for Myogenesis

**DOI:** 10.3390/ijms241512262

**Published:** 2023-07-31

**Authors:** Chuan-Che Wu, Wei-Cheng Chen, Wen-Po Hsiao, Kai-Fan Huang, Yi-Shiuan Liao, Huang-Bin Lin, Yi-Ju Wu, Chien-Han Kao, Shen-Liang Chen

**Affiliations:** Department of Life Sciences, College of Health Sciences and Technology, National Central University, Taoyuan 320317, Taiwan; jaywu929@gmail.com (C.-C.W.); love0961108532@gmail.com (W.-C.C.); x28520791@gmail.com (W.-P.H.); ncu.108801012@g.ncu.edu.tw (K.-F.H.); sandyliao.900614@gmail.com (Y.-S.L.); andy7391350@gmail.com (H.-B.L.); kathywu614@gmail.com (Y.-J.W.); sos456852sos@livemail.tw (C.-H.K.)

**Keywords:** muscle, peroxisome, mitochondria, myogenesis, catalase, *Pex3*

## Abstract

Mitochondria (MITO) and peroxisomes (PEXO) are the major organelles involved in the oxidative metabolism of cells, but detailed examination of their dynamics and functional adaptations during skeletal muscle (SKM) development (myogenesis) is still lacking. In this study, we found that during myogenesis, MITO DNA, ROS level, and redox ratio increased in myotubes, but the membrane potential (Δψm) and ATP content reduced, implying that the MITO efficiency might reduce during myogenesis. The PEXO number and density both increased during myogenesis, which probably resulted from the accumulation and increased biogenesis of PEXO. The expression of PEXO biogenesis factors was induced during myogenesis in vitro and in utero, and their promoters were also activated by MyoD. Knockdown of the biogenesis factors *Pex3* repressed not only the PEXO density and functions but also the levels of MITO genes and functions, suggesting a close coupling between PEXO biogenesis and MITO functions. Surprisingly, *Pex3* knockdown by the CRISPRi system repressed myogenic differentiation, indicating critical involvement of PEXO biogenesis in myogenesis. Taken together, these observations suggest that the dynamics and functions of both MITO and PEXO are coupled with each other and with the metabolic changes that occur during myogenesis, and these metabolic couplings are critical to myogenesis.

## 1. Introduction

Skeletal muscle (SKM) is the largest metabolic organ and constitutes approximately 35–40% of the adult body mass in mammals, and it is highly energy demanding because of its essential role in various physical activities, such as ambulation and various body movements [1]. Therefore, a proper energy supply is critical for the SKM, as well as the whole body’s health. Mitochondria (MITO) and peroxisomes (PEXO) are the major organelles involved in oxidative metabolism that generate ATP currency in the cell, and their biogenesis and functions are critically regulated by the transcriptional coactivator PGC-1α [2,3]. MITO are double-membraned organelles that carry extra-nuclear DNA genome (mtDNA), and they provide ATP currency through oxidative phosphorylation (OXPHOS) of the reducing equivalents FADH2 and NAD(P)H, which are mainly derived from glycolysis and the tricarboxylic acid (TCA) cycle, which occur in the cytoplasm and MITO matrix, respectively [4]. OXPHOS is performed by five complexes embedded in the MITO inner membrane, and electron leakage from the OXPHOS is a major source of reactive oxygen species (ROS) in the cell.

PEXO are single-membrane-bounded organelles that do not carry genetic material [5,6]. In addition to the β-oxidation of very long chain, unsaturated, and branched fatty acids, they can also metabolize carboxylates containing a 3-methyl or 2-hydroxy group via α-oxidation, whereby one-carbon shortened products can be passed onto the β-oxidation system [6]. Another important function of peroxisomes is the removal of harmful reactive oxygen species (ROS) concomitantly generated via oxidation of substrates in mitochondria and peroxisomes, which is performed by several antioxidant enzymes, including catalase, SOD1, and PRDX5.

Both mitochondria and peroxisomes are highly dynamic, and their number and morphology are regulated by the balance between biogenesis and autophagy (mitophagy and pexophagy, respectively) to meet physiological demands, such as exercise, in the SKM. Oxidative exercise induces MITO proliferation to promote oxidative respiration which, in turn, will increase the number of type-I myotubes in SKM, while a sedentary lifestyle or disuse antagonizes this process [7]. The fusion and fission of existing MITO are the two processes that generate new MITO, and both are mediated by members of the dynamin GTPase-related protein (DRP) family. Enhanced fusion creates MITO networks that are higher in membrane potential (Δψ_m_) and ATP production efficiency; on the contrary, a lack of fusion generates more vesicular MITO that are poor in efficiency (reviewed in [8]), probably due to increased mtDNA mutation and depletion [9].

The biogenesis of peroxisomes can be achieved through the fission of existing peroxisomes and de novo from ER- and MITO-derived membrane; regardless of the pathway, the import of matrix and membrane proteins is essential for biogenesis. Peroxisome matrix proteins carrying peroxisome targeting signal 1 or 2 (PTS-1/2) can be recognized by their cognate receptors, Pxe5 or Pex7, respectively, before being carried to the surface for translocation into peroxisomes [6]. Peroxisome membrane proteins (PMPs) can be recognized by cytoplasmic receptor Pex19, which docks onto Pex3, located on the peroxisome’s surface, to transfer PMPs to PEXO via the class I pathway. Pex3 is also recognized by Pex19 in the cytoplasm, and it is received by Pex16, located on pre-peroxisome’s membrane, via the class II pathway, and the newly integrated Pex3 recruits more Pex19, which carry PMPs, to generate mature PEXO. After docking, Pex19 is recycled back to the cytoplasm via both pathways. A recent study found that Pex3 and Pex16 are preferentially located on pre-peroxisome vesicles derived from MITO and ER, respectively, demonstrating the differential contribution of the membrane from both organelles [10]. Peroxisome proliferators, such as fenofibrate, stimulate peroxisome fission in which Pex11βs, which oligomerizes on elongated peroxisome surfaces, recruit fission machinery consisting of Mff, Fis1, and DLP1 to promote peroxisomal fission.

Although MITO and PEX perform independent roles in the metabolism, they collaborate in many metabolic processes, especially fatty acid β-oxidation (FABO) and ROS metabolism [11]. Fatty acids of short (<C8), medium (C8−C14), and long (C16−C20) chains are majorly metabolized in MITO. Peroxisomes prefer very long chain fatty acids (VLCFA, >C20) for oxidation but only digest them to C8−C6 before transferring them to MITO for further processing. Acetyl-CoA, generated from FABO in peroxisomes, is transported into MITO for further metabolism in the TCA cycle. ROS leaked from MITO can be handled by the scavenging systems in peroxisomes and cytoplasm. It will be interesting to determine how the functions and crosstalk of these two organelles are coordinated during myogenesis and the development of SKM.

All trunk and limb SKM cells in vertebrates are derived from progenitor cells residing in transient embryonic tissue, called somites, located on both sides of the neural tube [12]. Myogenic stem cells (MSCs) in somites are marked by the expression of *Pax3* and *Pax7,* and they become myoblasts after the expression of *Myf5* or *MyoD* [13]. Upon the stimulation of differentiation signals, myoblasts (*Myf5^+^* or *MyoD^+^*) start expressing *Myogenin* and *Mef2c* that cooperatively drive the terminal myogenic differentiation process. A later stage of muscle development, including alignment and maturation of myotubes, is regulated by Mrf4. The expression of *MyoD*, *Myf5*, *Myogenin*, and *Mrf4* is highly SKM-specific, and they are collectively called myogenic regulatory factors (MRFs). MRFs recognize a CANNTG *cis*-element (called E-box) with their basic helix–loop–helix (bHLH) DNA-binding domain, and they control the expression of most, if not all, SKM-specific genes [14]. Notably, Mrf4 is the dominant MRF in mature myofibers of postnatal muscles, while MyoD is essential to the activation of SKM stem cells, the satellite cells, and thus critical to the regeneration of damaged SKM [15].

Since SKM is a highly energy-demanding organ that is highly reliant on oxidative metabolism performed in MITO and PEXO, the coordinated regulation of both organelles should play critical roles in its development. However, the dynamics and functional changes of MITO and PEXO during this process have not been observed before. Here, the dynamics of both organelles were examined during myogenesis in vitro and in vivo/utero, and an interesting change in number and function was observed during myogenesis. Hopefully, unraveling the coupled regulation of both organelles will shed light on the complex mechanism of cell differentiation.

## 2. Results

### 2.1. MITO Efficiency Decreases during Myogenesis

**Figure 1:**
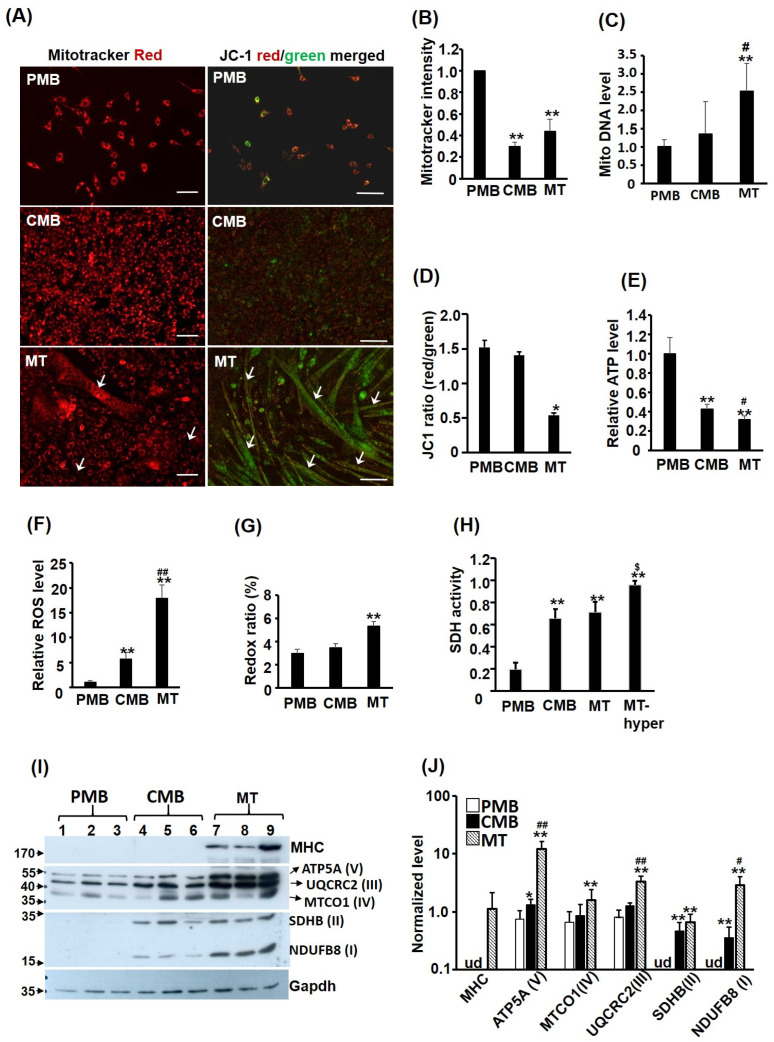
Mitochondrial efficiency declines during myogenesis. C2C12 myoblasts at proliferating myoblast (PMB), confluent myoblast (CMB), and differentiated myotube (MT) stages were treated with Mito Tracker-Red CMXRox (50 ng/mL, 30 min) or JC-1 (2 μM, 30 min) fluorescence dye and their MITO morphology is shown in the left and right panels of (**A**), respectively. Their fluorescence intensity was quantified via photometry and is shown in (**B**,**D**), respectively. Arrow: myotube. Genomic DNA was isolated from C2C12 cells of all stages, and their relative level (vs. PMB) of MITO DNA is shown in (**C**). The levels of ATP, ROS, and redox ratio ((FAD/(NADH + FAD) × 100) were measured in C2C12 myoblasts of different stages and the results are shown in (**E**–**G**). (**H**) The succinate dehydrogenase (SDH) activity of cells at different stages and in hypertrophic myotubes (MT-hyper) induced by insulin (20 nM) and LiCl (5 mM) was measured. Levels of key components of the OXPHOS complexes (in parenthesis) were examined via Western blot (**I**) in triplicates and the quantified results are shown in (**J**). Results are shown as mean ± SE and *n* = 3 for all assays in this figure. Scale bar: 100 µm. * *p* < 0.05 and ** *p* < 0.01 vs. PMB. ^#^ *p* < 0.05 and ^##^ *p* < 0.01 vs. CMB. ^$^ *p* < 0.05 vs. MT. ud: undetectable.

The mitochondrion is the most important energy producer and oxidative organelle in eukaryotic cells, so its functions and quantity should be critically regulated during myogenesis to meet the metabolic changes during this process. C2C12 myoblasts at proliferating myoblast (PMB), confluent myoblast (CMB), and differentiated myotube (MT) stages are treated with Mito Tracker-Red CMXRox (mitotracker) fluorescent dye to identify the MITO morphology and the fluorescence intensity to determine the inner membrane potential (Δψm). The cytoplasm of cells at all stages was full of red staining, suggesting extensive MITO distribution in the cytoplasm (Figure 1A, left panel and Supplementary Figure S1A). However, some myotubes were found to be mitotracker-free (comparing images of red and bright field, Supplementary Figure S1A). Their staining intensity was further characterized by fluorescence spectrophotometry, and we were surprised to find that mitotracker intensity dropped drastically from PMB to CMB but leveled up only slightly in myotubes (Figure 1B), suggesting a reduction in either the MITO amount and/or its potential across the inner membrane (Δψm). The MITO amount was examined via quantification of MITO DNA and a very significant increase was found in myotubes compared to that in PMB and CMB cells (Figure 1C), suggesting a decline in the MITO Δψm, instead of the MITO amount, during myogenesis.

A fluorescent dye more sensitive to the membrane potential Δψm is JC-1 dye, which is green in the cytoplasm but forms red aggregates in MITO in response to Δψm (Figure 1A, right panel and Supplementary Figure S1B). Therefore, the ratio of red/green fluorescence can serve as a very reliable indicator of Δψm. We found that the JC-1 ratio was similar between PMB and CMB, but reduced significantly in the myotubes, confirming the reduced Δψm in myotubes (Figure 1D). The reduced JC-1 ratio in myotubes was further examined using a microscope and camera with better resolution, and we found less JC-1 red aggregates in some myotubes, especially in hypertrophic myotubes (Supplementary Figure S1C), which might be the main reason for the reduced JC-1 ratio in myotubes. We also noted a gradually reduced ATP level but an increased reactive oxygen species (ROS) level during myogenesis (Figure 1E,F). These observations indicate a decline in MITO efficiency during myogenesis. The redox ratio (FAD/(NADH + FAD)) was also significantly increased in myotubes (Figure 1G), suggesting a higher ATP demand causing increased oxidative phosphorylation (OXPHOS) in the myotubes [16]. To confirm the increased OXPHOS, succinate dehydrogenase (SDH) activity in the complex II was examined and it was significantly increased from PMB to CMB, myotubes, and hypertrophic myotubes (Figure 1H). Furthermore, the levels of key components in the OXPHOS complexes were all up-regulated during myogenesis (Figure 1I,J), suggesting that reduced MITO efficiency is not due to reduced levels of OXPHOS complexes.

### 2.2. Peroxisome Number Is Increased during Myogenesis

**Figure 2:**
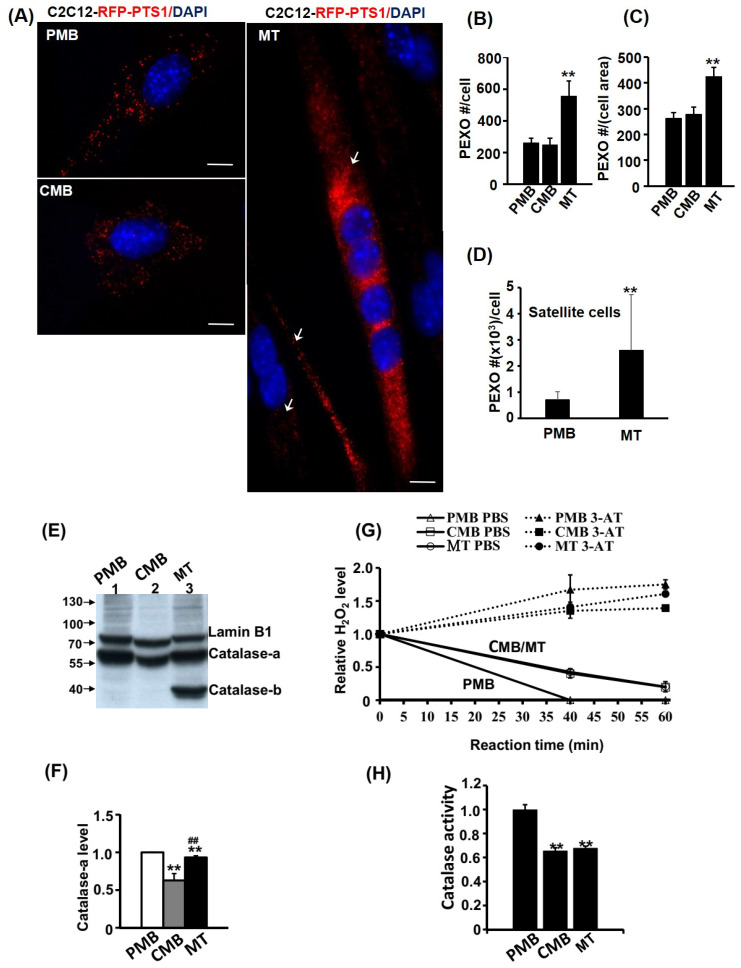
PEXO number increases but function declines during myogenesis. The dynamics of PEXO number during myogenesis of C2C12 myoblasts stably over-expressed with *RFP-PTS1* (C2-RFP-PTS1) was followed and counted. The morphology is shown in (**A**) and their PEXO number per cell and density (normalized by cell size) is shown in (**B**,**C**), respectively. The PEXO number was counted in 180 cells of PMB and CMB stages and in 60 myotubes. Arrow: myotube. PEXO number during satellite cell differentiation was detected by PMP70 immunofluorescence and their numbers in PMB (*n* = 21) and MT (*n* = 13) stages are shown in (**D**). Catalase levels in total lysate (50 µg) were determined via Western blot using Lamin B1 as the loading control (**E**) and the quantified results are shown in (**F**). (**G**) PEXO function was measured via the removal of H_2_O_2_ in a time course and the specificity was confirmed based on the activity inhibited by the Catalase inhibitor 3-amino-1,2,4-triazole (3-AT, 20 mM). The normalized Catalase activity at 40 min reaction time is shown in (**H**). *n* = 3 for (**F**–**H**). Scale bar: 10 µm. ** *p* < 0.01 vs. PMB. **^##^** *p* < 0.01 vs. CMB.

Peroxisomes (PEXO) are also important energy suppliers in the mature myocytes, so it is important to examine their functional and quantitative dynamics during myogenesis. Using C2C12 myoblasts stably carrying the *RFP-PTS1* expression vector (C2-RFP-PTS1), the numeric change of PEXO was characterized. We found that PEXO number remained constant in PMB and CMB cells but was increased about twofold in myotubes (Figure 2A,B). When normalized with the area of each cell (myotubes are much larger), the density of PEXO remained higher in myotubes than those in previous stages (Figure 2C). As C2C12 are cells kept in culture long term, their physiology might be subtly different from those in vivo. To avoid this possible artifact, primary satellite cells were isolated and their PEXO dynamics during myogenesis were observed via immunofluorescence detection of PMP70, a PEXO membrane protein. Interestingly, more PEXO were found in PMB satellite cells compared with C2C12 and their number increased as they became myotubes (Figure 2D and Supplementary Figure S1D). Therefore, an increased PEXO number during myogenesis is a common phenomenon shared by both C2C12 and satellite cells, but whether the increased PEXO number contributes to myogenic differentiation has yet to be examined in detail.

Removing free radicals in the cells using the enzyme Catalase is one of the important functions of PEXO, so the protein level and activity of Catalase during myogenesis was also examined. The Catalase protein (about 65 kd, designated as Catalase-a here) level declined slightly in CMB cells but returned to a higher level in myotubes (Figure 2E,F). However, a lower band of Catalase (designated as Catalase-b here) at around 40 kd was found only in myotubes, suggesting that some Catalase might be processed uniquely or induced for degradation in myotubes. It was of interest to know whether the change in Catalase protein level could reflect its activity. Using H_2_O_2_ metabolism as an indicator of Catalase activity, a significant decrease in CMB and MT cells was observed compared with the PMB cells (Figure 2G,H). The reduced Catalase activity but increased PEXO number/density in myotubes suggest that the PEXO in myotubes are functionally deficient.

### 2.3. PEXO Is Accumulated during Myogenesis Due to Biogenesis and Slow Removal

**Figure 3:**
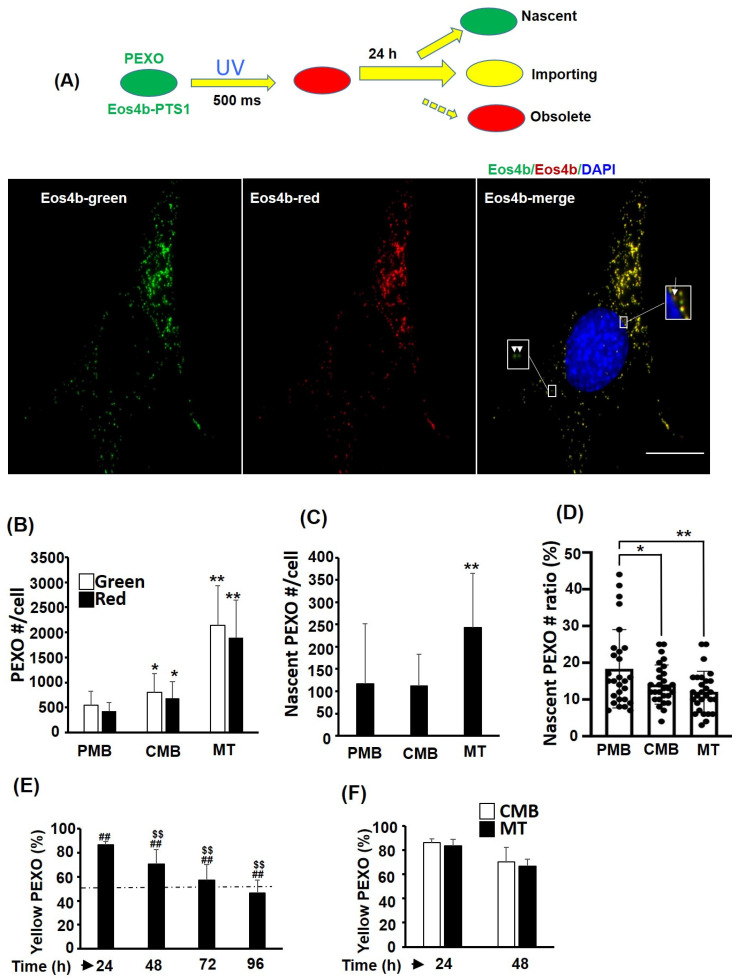
PEXO biogenesis is increased during myogenesis. (**A**) Schematics showing the color change of Eos4b-PTS1-hosting PEXO before and after UV exposure (about 500 milliseconds), and various groups of PEXO can be identified by their color after 24 h (top panel). Pure green or red PEXO represents nascent or old/obsolete PEXO. Yellow PEXO are functional and import new Pex proteins. Representative images of C2C12 myoblasts stably over-expressed with Eos4b-PTS1 (C2-*Eos4b-PTS1*) are shown at the lower panel. Arrow: red/orange PEXO; Arrow head: green PEXO. The numbers and ratios of various PEXO in C2-*Eos4b-PTS1* during myogenesis were counted, and the total number is shown in (**B**) and the number and ratio of nascent PEXO are shown in (**C**,**D**), respectively. *n* = 28 for (**B**–**D**). (**E**,**F**) The ratios of yellow PEXO in cells of CMB and MT stages were calculated at various times (24–96 h) post UV exposure, *n* = 5. Dashed line indicates 50%. Scale bar: 20 µm. * *p* < 0.05 and ** *p* < 0.01 vs. PMB. ^##^ *p* < 0.01 and ^$$^ *p* < 0.01 vs. time zero and 24 h, respectively.

The reduced Catalase activity in myotubes implies that PEXO in myotubes might be aged or less efficient. Therefore, the dynamics of PEXO during myogenesis were examined based on the Eos4b-PTS1 fluorescent protein, which changed color from green to red if exposed to UV light transiently (Figure 3A). C2-Eos4b-PTS1 stable clone cells were exposed to UV for a short period of time (500 milliseconds) and the numbers of green, red, and yellow PEXO were counted 24 h later. Theoretically, pure green and red PEXO represent nascent and obsolete PEXO, respectively (Figure 3A, insets). Yellow PEXO are functional and continue importing new PEXO proteins during the 24 h. The green and red PEXO numbers were counted at different myogenic stages of C2-Eos4b-PTS1 cells, and a stepwise increase in PEXO number per cell was observed (Figure 3B). Most PEXO in C2-Eos4b-PTS1 cells were yellow regardless of stage, suggesting they were functional and receiving new matrix proteins. However, the nascent PEXO number per cell was only marginally increased in myotubes, but the ratio of nascent PEXO was significantly reduced in the myotubes due to a significant increase in the PEXO number per cell at this stage (Figure 3B–D). Furthermore, pure red PEXO was difficult to identify in cells of all stages and only some orange/red ones could be found (Figure 3A, insets), implying that obsolete PEXO should be removed efficiently at all stages. The half-life of PEXO in C2-Eos4b-PTS1 cells at CMB stage was estimated by exposing cells to UV for a short period of time and followed the yellow (red pre-existing PEXO with newly imported green Eos4b-PTS1) PEXO for 4 days (Figure 3E). A gradually reducing trend was found and the half-life fell between 72 and 96 h (about 86 h), indicating accumulation and slow removal of pre-existing PEXO during myogenesis. The PEXO removing rate was compared between CMB and MT cells and no significant difference was found (Figure 3F). These observations suggest that the majority of PEXO in myogenic cells of all stages import matrix protein (yellow) to perform their functions, but the ratio of nascent PEXO in myotubes is slightly lower than that in cells of other stages, which might be part of the reason for the lower PEXO efficiency in myotubes.

### 2.4. PEXO Biogenesis Is Induced during Myogenesis

**Figure 4:**
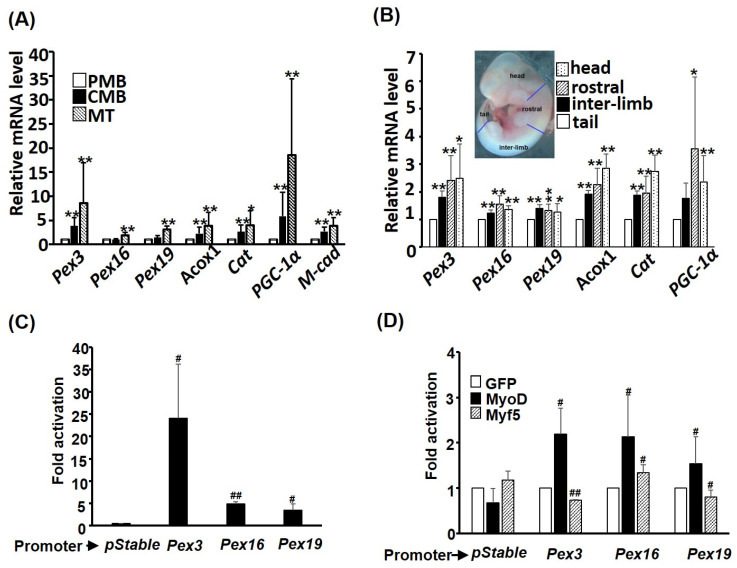
PEXO biogenesis factors are induced during myogenesis. The expression levels of PEXO biogenesis factors in C2C12 myoblasts (**A**) and embryos (**B**) were examined via qRT-PCR. The inset in (**B**) indicates the various regions in an embryo (11.5 dpc) isolated to determine the gene expression level. * *p* < 0.05 and ** *p* < 0.01 vs. PMB in (**A**) or tail in (**B**). The promoters of *Pex3, Pex16*, and *Pex19* were cloned into luciferase reporter pStable-luc and their basal activity in differentiating C2C12 cells is shown in (**C**) and their regulation by MRFs is shown in (**D**). The activity of pStable-luc in (**C**) or the activity of each promoter co-transfected with GFP expression vector was set as 1-fold activation. Results are shown as mean ± SE and *n* = 3 for all assays in this figure. ^#^ *p* < 0.05 and ^##^ *p* < 0.01 vs. pStable in (**C**) or GFP in (**D**). *Cat: Catalase; M-cad: M-cadherin*.

The increase in nascent PEXO implies the activity of de novo biogenesis of PEXO might be enhanced in myogenesis. To confirm this hypothesis, the expression level of the key receptors/binding proteins, *Pex3, Pex16,* and *Pex19*, in this process was examined during C2C12 myogenesis, and we identified an increased trend of these factors, especially in myotubes (Figure 4A). Therefore, the increased nascent PEXO in myotubes observed above (Figure 3C) is supported by enhanced de novo biogenesis. This observation was further examined in embryonic myogenesis by comparing their expression levels in tail, interlimb, and rostral somites. Somitogenesis in vertebrates proceeds from rostral to tail, so rostral somites are much more mature than those in the tail. During the maturation/development of somites, an increased trend of de novo biogenesis factors and other PEXO factors was observed, which further strengthened the conclusion that de novo biogenesis is enhanced during myogenesis (Figure 4B). The mRNA levels of *Acox1* and *Catalase*, two key enzymes in PEXO, were also found to be increased during myogenesis in vitro and in vivo. (Figure 4A,B).

The increased expression of *Pex3. Pex16,* and *Pex19* during myogenesis suggests that their expression might be activated by myogenic regulatory factors (MRFs). Promoters of these factors were cloned and examined via transient promoter assay to answer this question, and strong activity of these promoters, especially *Pex3*, was observed (Figure 4C). Co-transfection of *MyoD* significantly increased the activity of all three promoters, while *Myf5* slightly repressed *Pex3* and *Pex19* but increased *Pex16* promoter activity (Figure 4D). Taken together, these observations suggest that MyoD activates the expression of de novo factors at the transcriptional level to support PEXO de novo biogenesis during myogenesis.

### 2.5. Pex3 Knockdown Reduces PEXO Biogenesis

**Figure 5:**
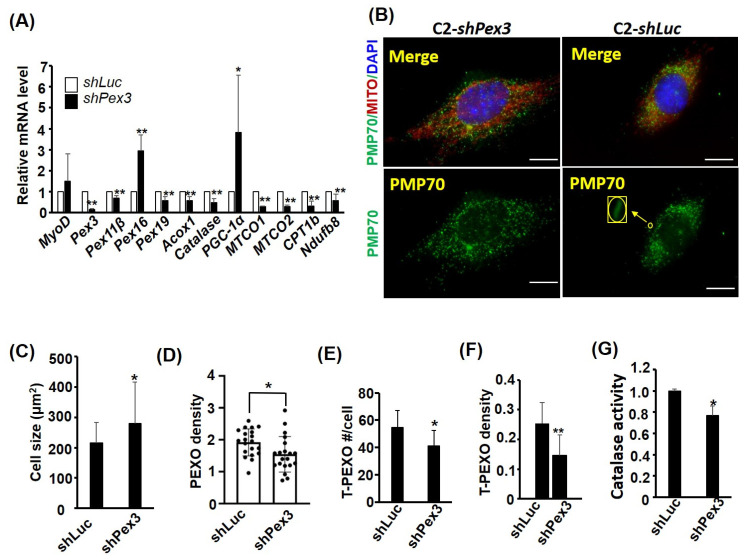
*Pex3* knockdown reduces PEXO density. The expression of *Pex3* in C2C12 cells was stably knocked down (KD) by lentiviral vector-expressed *shRNA* to generate *Pex3* KD stable clones (C2-*shPex3*). (**A**) The expression levels of PEXO and MITO factors in the C2-*shPex3* and –*shLuc* myoblasts (CMB) were examined via qRT-PCR with the level in the C2–*shLuc* cells set as 1-fold, *n* = 3. (**B**) PEXO and MITO in the C2-*shPex3* and the control cells *(*C2-*shLuc)* were detected via immunofluorescence (with antibody against PMP70) and Mito Tracker-Red CMXRox. Scale bar: 10 µm. Their cell size, PEXO number and density (#/µm^2^) are shown in (**C**–**E**). *n* = 20 for (**C**–**F**). The number and density (#/µm^2^) of tubular PEXO (T-PEXO, shown as an inset in (**B**)) in both cells, are shown in (**E**,**F**). Their Catalase activity is shown in (**G**), *n* = 3. T-PEXO: tubular PEXO. * *p* < 0.05 and ** *p* < 0.01 vs. C2-*shLuc* cells. All assays were performed on confluent myoblasts.

The increased de novo biogenesis of PEXO during myogenesis raises the question whether it is essential to myogeneis. Among the biogenesis factors, *Pex3* showed the strongest induction in myotubes; therefore, a lentiviral *shRNA* knockdown approach was adopted to clarify how important *Pex3* is to PEXO biogenesis in myoblasts. Expression profiling of the *Pex3* knockdown stable cells, C2-*shPex3*, at CMB stage confirmed a reduction in *Pex3*, as well as other PEXO factors’ mRNA levels (Figure 5A). However, the levels of both *Pex16* and *PGC-1α* were significantly increased, suggesting pathways compensating for the reduced *Pex3* level have been activated. The levels of several OXPHOS factors were also examined and we were surprised by their uniform reduction, which suggests a close cooperation between PEXO and MITO, and indicates that a defect in one organelle might induce malfunction in another. Surprisingly, a larger cell size was induced by the *Pex3* knockdown but no difference in the number of PEXO per cell between control and the *Pex3* knockdown cells was observed (Figure 5B,C and Supplementary Figure S2A). Therefore, the density of PEXO per area was reduced in the CMB C2-*shPex3* stable cells (Figure 5D). Further examination of the PEXO morphology revealed less tubular PEXO in the C2-*shPex3* stable cells, no matter their number, ratio, or density (Figure 5E,F, and Supplementary Figure S2B). As tubular PEXO are those growing precursors for fission into the normal vesicular PEXO, their reduction suggests the compromised transportation of PMPs and membrane to existing PEXO for their growth and fission in the C2-*shPex3* cells. The compromised biogenesis also reduced the Catalase activity (Figure 5G), suggesting its importance in maintaining the function of PEXO.

### 2.6. Pex3 Knockdown Compromises MITO Functions

**Figure 6:**
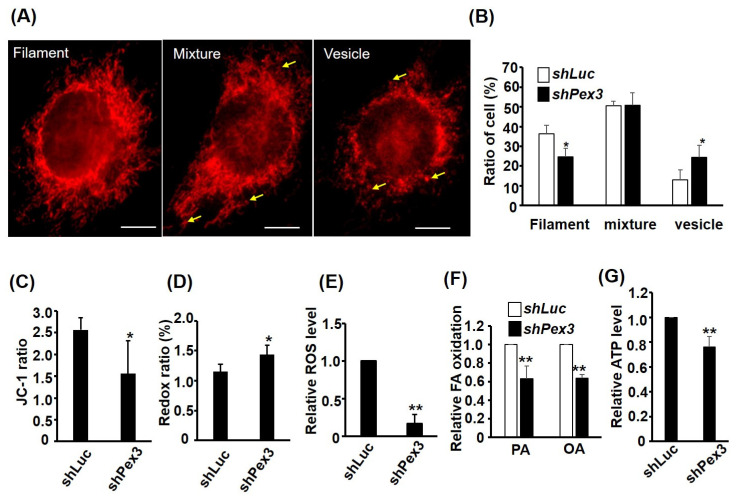
MITO functions are compromised in *Pex3* knockdown cells. The morphology of MITO in C2-*shPex3* and –*shLuc* myoblasts was viewed by Mito Tracker-Red CMXRox staining (**A**) and the ratio of cells (*n* = 75) with MITO morphology in filament, vesicle, and mixed shapes is shown in (**B**), *n* = 3. Scale bar: 20 µm. Arrow: vesicle MITO. MITO membrane potential was detected with JC-1 staining and the ratio (red/green) is shown in (**C**), *n* = 5. Both redox ratio (FAD/(FAD + NADH) and ROS were measured via spectrophotometry and are shown in (**D**,**E**). Fatty acid (FA) β-oxidation was measured via oxidation of ^3^H-labeled palmitic acid (PA) and oleic acid (OA), and the relative oxidation ability is shown in (**F**). The relative ATP level is shown in (**G**). *n* = 6 for (**D**) and *n* = 3 for (**E**–**G**). * *p* < 0.05 and ** *p* < 0.01 vs. C2-*shLuc* cells. All assays were performed on confluent myoblasts.

The defected PEXO growth in the C2-*shPex3* cells prompted us to examine their PEXO and MITO functions. Previous studies have found that MITO functions can be reflected by their morphology, in which filamentary and vesicular MITO represent high and low efficient functions, respectively (reviewed in [8]). Using Mito Tracker-Red CMXRox staining, we found that cells with filament MITO were reduced but those with vesicle MITO were increased in the C2-*shPex3* cells, suggesting compromised MITO function (Figure 6A,B), which was further confirmed by the reduced Δψ_m_ as shown by the JC-1 ratio (Figure 6C). We also found that redox ratio (FAD/(NADH + FAD)) was increased by *Pex3* knockdown, implying an accumulated FAD level caused by slow flux through the oxidative phosphorylation chain in the MITO (Figure 6F). This hypothesis was further demonstrated by the reduced ROS, fatty acid β-oxidation, and ATP levels in the C2-*shPex3* cells (Figure 6E–G). Taken together, *Pex3* knockdown affected not only PEXO but also MITO functions in the myoblasts.

### 2.7. Pex3 Over-Expression Enhances PEXO Biogenesis

**Figure 7:**
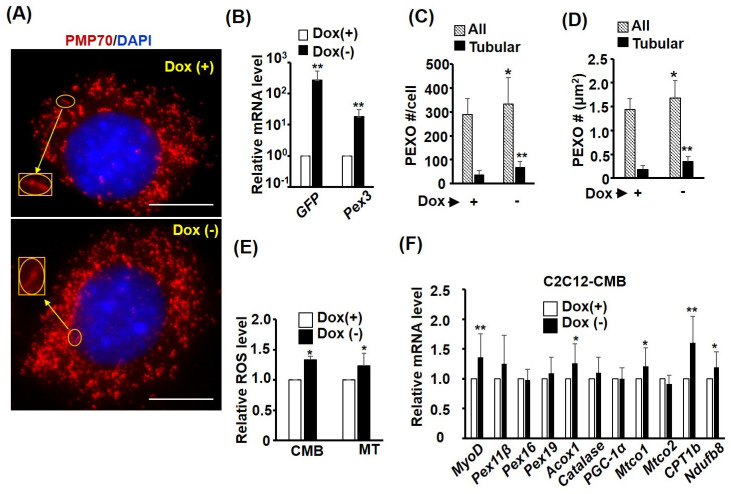
*Pex3* overexpression enhances PEXO biogenesis and density. A stable clone, *C2-tTA-Pex3*, overexpressed with *GFP* and *Pex3* under a bi-directional *Tet-off* system was generated to allow doxycycline (Dox) repressed expression. PEXO morphology in these cells was detected via PMP70 immunofluorescence (**A**) and the levels of *GFP* and *Pex3* in the presence and absence of doxycycline (Dox (+/−), 25 ng/mL) were examined via qRT-PCR (**B**), *n* = 3. Their PEXO number and density under Dox (+/−) are shown in (**C**,**D**). The insets in (**A**) show higher magnification view of tubular PEXO (T-PEXO). *n* = 30 for (**C**,**D**). Their ROS levels at CMB and MT stages are shown in (**E**), *n* = 3. (**F**) The expression levels of PEXO and MITO factors in the *C2-tTA-Pex3* myoblasts (CMB) were examined via qRT-PCR with the level in the Dox (+) cells set as 1-fold (*n* = 3). Scale bar: 10 µm. * *p* < 0.05 and ** *p* < 0.01 vs. Dox (+).

A *Tet-off* system was employed to over-express *Pex3* stably in the C2C12 cells (*C2-tTA-Pex3*), in which *GFP* and *Pex3* were both highly expressed after removal of doxycycline (Dox (−)); however, the relative folds of increase in the *Pex3* level (17 folds) were lower than that (283 folds) of *GFP* due to its high endogenous level (Figure 7A,B). The over-expressed *Pex3* level increased the PEXO number marginally but significantly, either in number or in density (Figure 7C,D), suggesting that an increased *Pex3* level is beneficial to PEXO biogenesis in myoblasts. This notion could also be demonstrated by the highly increased number of tubular PEXO, no matter the number or density (Figure 7C,D). These observations suggest that PEXO biogenesis in myoblasts is sensitive to the *Pex3* level so more tubular PEXO can be seen if the *Pex3* level is increased. It has been reported that an increased *Pex3* level can increase pexophagy activity, which provides the reason for the marginal increase in the PEXO number after *Pex3* over-expression [17]. We also found the ROS levels were increased by *Pex3* over-expression in cells at CMB and MT stages, demonstrating the critical role of PEXO biogenesis in ROS regulation (Figure 7H). Gene expression analysis of PEXO and MITO factors in CMB cells revealed increased levels of *CPT1b, Mtco1, Ndufb8,* and *Acox1*, but not other factors, suggesting they are the factors most sensitive to PEXO biogenesis (Figure 7F).

### 2.8. The Function of Pex3 Is Important for Myogenesis

**Figure 8:**
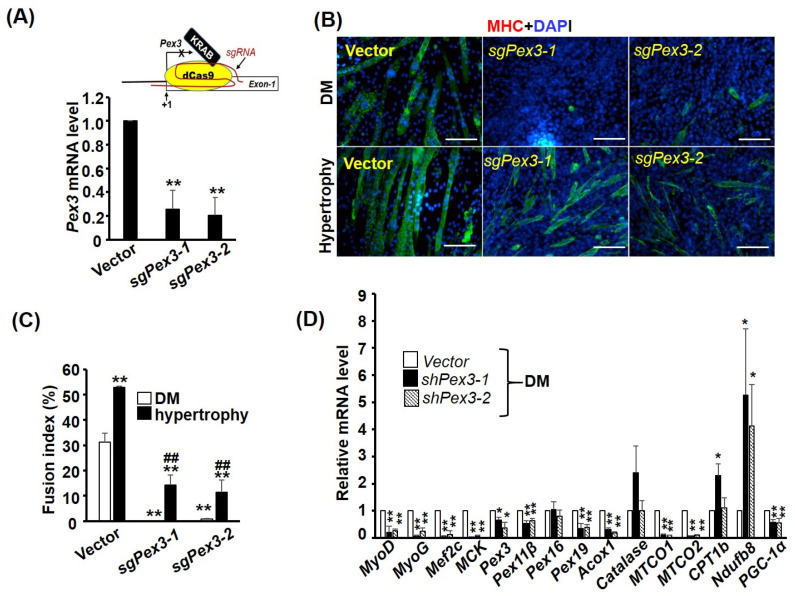
*Pex3* is required for myogenesis. (**A**) Schematics representing the CRISPRi approach are shown in the top panel. KRAB-dCas9 fusion protein was guided by *sgRNA* (*sgPex3-1* or *sgPex2-2*) to the region around *Pex3* gene initiation site (+1) to suppress its transcription. The target sites for *sgPex3-1* and *sgPex3-2* are −14~+6 and −11~+9, respectively. The knockdown efficiency of C2C12 stable clones (C2*-sgPex3*) carrying either sgRNA was examined via qRT-PCR and is shown in the bottom panel (*n* = 3). (**B**) Images of vector control and C2*-sgPex3-1* and *-2* stable clones after 4 days in differentiation medium (DM, upper panel) or hypertrophy medium (DM with insulin (25 nM) and LiCl (5 mM)) for inducing hypertrophy (lower panel). Their fusion indexes (*n* = 9) are shown in (**C**) and the gene expression pattern of C2-*sgPex3* cells in DM was examined via qRT-PCR (**D**), *n* = 3. Scale bar: 20 µm. * *p* < 0.05 and ** *p* < 0.01 vs. vector control; ^##^ *p* < 0.01 vs. hypertrophy vector control.

As the expression of most PEXO factors is increased during myogenesis (Figure 4), it is of interest to verify whether the increased *Pex3* level is essential for myogenesis. Unfortunately, although the lentiviral *shRNA* system allowed us to set up stable clones to analyze its effect, this system cannot be used to reveal the knockdown effect on myogenesis, as it represses myogenic differentiation (data not shown). Furthermore, myoblasts struggle to transfect *shRNA* oligomers and the half-life of *shRNA* oligomers in the cells is too short for the period (4–6 days) of myogenesis assay. After testing several expression systems, we found the CRISPRi system can knock down gene expression efficiently and, at the same time, preserve the myogenic ability (Figure 8A). Using two independent *sgRNAs* (targeting −14~+6 and −8~+12 of *Pex3* gene, respectively) expressed by the CRISPRi system, we found the level of *Pex3* was significantly reduced in stable clones, C2-sg*Pex3*, over-expressed with either *sgRNA* (Figure 7A). The myogenesis of C2-sg*Pex3* stable cells was significantly inhibited as shown by their morphology and fusion index (Figure 8B,C). Interestingly, the myogenesis of C2-sg*Pex3* cells could be partially rescued by the hypertrophic signals, insulin and LiCl, even though their fusion index were still lower than that of vector control cells, indicating the myogenic signaling pathways in C2-sg*Pex3* cells are highly repressed.

The gene expression pattern of C2-sg*Pex3* cells was further analyzed with qRT-PCR and the levels of myogenic factors *MyoD*, *MyoG*, and *Mecf2* and maturation marker gene *muscle creatine kinase (MCK)* were dramatically reduced (Figure 8D), demonstrating the repression of myogenesis. *Pex3* knockdown also reduced the levels of most PEXO-associated genes examined, especially *Pex19* and *Acox1*. Surprisingly, for MITO-associated genes, the levels of MITO-encoded *MTCO1* and *MTCO2* were drastically repressed but those encoded in the nucleus but functioning in MITO, such as *Ndufb8*, were significantly increased. The transcriptional coactivator *PGC-1α* functions both in the nucleus and in MITO, and its level was also reduced in C2-sg*Pex3* cells. Taken together, *Pex3*-knockdown induced dramatic alteration in the expression of both PEXO and MITO-associated genes, which in turn might repress myogenic differentiation by repressing the expression and function of myogenic genes. It will be interesting to identify the mechanism connecting organelle biogenesis and myogenesis, and we hope we can decipher it in the near future.

## 3. Discussion

### 3.1. Functional Decline of MITO and PEXO in Myotubes

Although SKM is the largest metabolic organ in our body and is highly energy-demanding, the dynamics and functional changes of these two energy-supplying organelles, MITO and PEXO, have seldom been documented during its development. Here, we followed their dynamics carefully according to various approaches and concluded that the biogenesis of both organelles was enhanced during myogenesis, which was supported by the increased mtDNA level and PEXO number in myotubes (Figure 1, Figure 2 and Figure 3). The increased biogenesis of PEXO was also evidenced by the gradually increasing levels of biogenesis factors, including *Pex3, Pex16,* and *Pex19,* during myogenesis in vitro and in utero. Intriguingly, although enhanced MITO functions were suggested by the increased SDH activity and OXPHOS complexes levels in CMB and MT cells, other metabolic parameters/indexes, such as reduced JC-1 staining and ATP level but increased ROS level and redox ratio, suggested compromised metabolic functions in myotubes. Reduced Catalase activity and fatty acid oxidation in myotubes also demonstrated compromised PEXO functions (Figure 2). Therefore, although higher numbers of MITO and PEXO are found in the myotubes, they are less functional and efficient.

The exclusion of JC-1 red aggregates from some myotubes, especially hypertrophic ones, was a surprising but meaningful discovery (Supplementary Figure S1C), which implies that either MITO membrane potential is disrupted or JC-1 aggregation is prevented by unknown means in these myotubes. Since the green image penetrated these myotubes very well, the lack of JC-1 red aggregates should not be caused by poor permeability of JC-1 dye. The lower ATP content but higher ROS and redox ratio suggest a leaky ETC across MITO inner membrane. Confirming the leaky ETC and its mechanism in myotubes should be the first priority in understanding metabolic adaptations during myogenic differentiation.

A discrepancy between the Δψm of CMB cells measured using Mitotracker and JC-1 was found (Figure 1B,D). Currently, we are not sure about the cause of this discrepancy, but it might be caused by the differential penetration of Mitotracker into cells of different stages. Since JC-1 measures the Δψm based on the red/green ratio, differential penetration rates will not cause a difference in the Δψm level. However, since Mitotracker and JC-1 are both fluorescent dyes, detecting the Δψm using a non-fluorescent dye-based method, such as a bioluminescent probe [18], should confirm the Δψm in the CMB cells.

The lower efficiency of PEXO in myotubes might be the result of the extended lifespan of obsolete ones (Figure 3), which might be caused by the delayed autophagy of PEXO (named pexophagy). Most peroxisomes (about 80%) are degraded through pexophagy [19], which is triggered by Pex14, a component of the Pex5 PTS-1 receptor docking complex, and ubiquitinated peroxisome membrane proteins (Ub-PMPs), such as Pex5 and PMP70 [20,21,22]. The ubiquitination of PMPs is majorly mediated by Pex2, an E3 ubiquitin ligase in the Pex2/Pex10/Pex12 translocation complex, which then recruits the autophagic adaptor p62/NBR1 and receptor LC3 to initiate pexophagy [23]. During starvation, Pex14 can directly interact with LC3-II, the phosphatidylethanolamine conjugated and active form of LC3, to activate pexophagy. Currently, studies on the pexophagy in SKM under various physiological conditions are very scarce, and it is very interesting to know whether it is compromised in myotubes and how will that affect PEXO functional efficiency.

The identification of the lower molecular weight Catalase in myotubes (Figure 2E) suggests that (1) a unique mRNA splicing variant might be produced in myotubes, (2) protein might be processed differently in myotubes, or (3) Catalase is targeted for efficient degradation. The antibody used here targets amino acids 471–503 of the Catalase protein, so the lower band should be a C-terminal fragment produced by one of the possible pathways mentioned above. Interestingly, the PCR primers used here target 147–313 bp of the mRNA (2551 bp in length), so the increased mRNA level seen in myotubes (Figure 4) should not include any C-terminal-only variants. A primer set targeting the C-terminal region should reveal whether a C-terminal variant exists in myotubes.

### 3.2. Regulation of PEXO Biogenesis Factors by MRFs

The increasing trend of PEXO numbers during myogenesis suggests that PEXO biogenesis might be regulated by the master regulators of myogenesis, myogenic regulatory factors (MRFs), as they control most aspects of myogenesis. Our previous studies have found that the promoter of *PGC-1α* gene, the master regulator of oxidative metabolism, is directly activated by all MRFs, and participation of P/CAF coactivator is required for this activation but can be repressed by the transcriptional repressor Bhlhe40 [24]. Strong protein–protein interaction between Bhlhe40 and PGC-1α was identified and it suggested coactivation of oxidative genes by PGC-1α was repressed by Bhlhe40 during myogenesis [25]. Follow-up studies have confirmed the importance of PGC-1α and Bhlhe40 interaction in the oxidative metabolism, especially in the PEXO and MITO functions [26]. The direct activation of *Pex3*, *Pex16*, and *Pex19* promoters by MyoD suggests that their regulation during myogenesis might be similar to that of *PGC-1α,* and it will be interesting to reveal the involvement of PGC-1α and Bhlhe40 in their activation by MyoD and other transcription factors, such as PPARδ.

The changes in the expression of myogenic genes during myogenesis are usually very dramatic and can reach a few hundred folds for some structure genes, such as *M-cadherin* and MHC [27]. However, only a few folds were seen here with the PEXO biogenesis factors (Figure 4), which raises concerns about how close their association with myogenesis is. The difference in the fold of change between these groups of genes might have something to do with their basal expression level. For instance, the basal expression level of *PGC-1α* is very limited (ΔCt is about 14–15 vs. m36b4) in this study, but the basal expression levels of PEXO biogenesis factors are relatively high (ΔCts are about 7–8 vs. m36b4). Therefore, a one-fold increase in the expression of these factors means a large number of transcripts and proteins must be produced in extra to achieve this increase, so their increase in expression is not as dramatic as that of *PGC-1α*, since the basal level is high and a few folds’ increase might be enough to couple with the metabolic changes. The promoter activity induced by MyoD is similar to their expression changes for *Pex16* and *Pex19*, but weaker than that of *Pex3* expression in utero and in vitro, suggesting *Pex3* expression might be targeted by extra factors, such as PPARs and PGC-1α, during myogenesis.

### 3.3. Coupling of MITO and PEXO Functions

Among these biogenesis factors, *Pex3* showed the highest level of induction and basal promoter activity during myogenesis (Figure 4), so it should be the factor most sensitive to the myogenic differentiation signals. Therefore, its level should be important to the activation of PEXO biogenesis during myogenesis, and the repression of myogenesis in C2-*sgPex3* cells has soundly demonstrated the importance of PEXO biogenesis in myogenesis (Figure 8). *Pex3* mutation was found to be the cause of peroxisome biogenesis diseases (PBD) of patients in the complementation group 12, in which no PEXO was detected and some PEXO proteins, such as Pex12, -13- and -14, were mis-localized to MITO [28]. Thus, *Pex3* loss-of-function mutations might affect not only the function of PEXO but also that of MITO. As tubular PEXO are the intermediate products of biogenesis [29]; their reduction in number induced by *Pex3* knockdown reflected the critical role of Pex3 in SKM PEXO biogenesis (Figure 5). It will be interesting to clarify whether PEXO proteins are mis-localized to MITO and how much this will affect MITO function.

The quality of MITO is regulated by several pathways and severely damaged MITO are removed via mitophagy (autophagy of MITO), in which MITO are wrapped in double-membrane vesicles, known as autophagosomes, and delivered to lysosomes for degradation. Initially, MITO damage stabilizes PINK1 kinase on the outer membrane which phosphorylates Mfn2 to serve as a receptor for the cytosolic E3-ubiquitin ligase Parkin. Parkin interacts with several autophagy-related proteins to promote MITO removal [30]. Mitophagy has been demonstrated as an essential event for remodeling MITO functions and morphology during myogenesis, in which MITO fragmentation is triggered by mitophagy generating vesicular MITO in the early stage but is replaced by a filamentary MITO network due to enhanced fusion at the late stage [31]. As *Pex3* knockdown enhances the number of cells with vesicular MITO (Figure 6), this suggests that the progression of myogenic differentiation might be held up at the early stage due to accumulation of vesicular MITO. Therefore, it will be interesting to determine whether the PINK1/Mfn2/Parkin pathway is activated by the *Pex3* knockdown to result in excessive mitophagy.

It is well known that MITO and PEXO are coupled in many metabolic functions, such as the β-oxidation of fatty acids. The interplay/dependence of these two organelles can be clearly described in conditions in which either organelle is defective. In the most frequent peroxisomal disorder X-linked adrenoleukodystrophy (X-ALD), VLCFAs (>C20) are accumulated to toxic level and incorporated into the MITO membrane, which leads to electron leakage from inner membrane and more superoxide and hydrogen peroxide production [32]. However, it was unknown before this study whether a reduction in PEXO biogenesis could change the efficiency of MITO functions before this study. The dramatic changes in MITO functions, including fatty acid β-oxidation, Δψ_m_, ROS level, and redox ratio, caused by *Pex3* knockdown implies that the coupling between these two organelles, especially in SKM, is tighter than expected (Figure 6). These observations also suggest that PEXO might play the role of an upstream regulator for MITO. Future studies should clarify whether this regulation is mediated by accumulated PEXO metabolites or via activation/repression of MITO regulatory factors, such as PGC-1α, NRFs, and ERRα, by PEXO factors. The up-regulation of *PGC-1α* amid reduced MITO functions and gene expression in C2-*shPex3* cells suggests that its function might be compromised in C2-*shPex3* cells and its up-regulation might be a compensatory response for the compromised function (Figure 5A).

### 3.4. Role of PEXO in Cellular ROS Level

It is normal to see ROS level increases during cell differentiation, as more oxidative metabolism is employed to generate ATP efficiently [33,34,35]. PEXO is a ROS generator and also a ROS sink, as it participates in the oxidation of many substrates and harbors many enzymes, such as Catalase and SODs, for the removal of H_2_O_2_ and other ROS. The ROS level was reduced in *Pex3* knockdown cells but increased in *Pex3* over-expressed cells (Figure 7G), demonstrating the critical role of PEXO in the regulation of cellular ROS levels. As the ROS level changed in negative correlation with PEXO biogenesis in *Pex3* knockdown or over-expressed cells, this suggests that PEXO generates more ROS than it can remove and its role of ROS producer is more prominent than the role of ROS sink. However, as PEXO and MITO are functionally coupled together, this negative correlation might be indirectly caused by the reduced functions of MITO. Future studies employing tools for detecting MITO- and PEXO-specific ROS might resolve the source of ROS after the *Pex3* expression level is changed. MitoSOX Red is a MITO-specific ROS fluorescent dye and should be an excellent tool for detecting the ROS source in *Pex3* knockdown or over-expressed cells [36]. Currently, there is no PEXO-specific dye for detecting ROS; however, roGFP2-PTS1 localized to PEXO can be used as ROS probe. This protein has two different and redox-dependent excitation peaks at 405 nm and 488 nm, and one single emission peak at 510 nm. While cysteine oxidation results in an increase in the 405 nm excitation peak, cysteine reduction increases excitation at 488 nm. Therefore, the ratio between emission at 510 nm resulting from excitation either at 488 nm or 405 nm can be used as an indicator of the relative amount of oxidized/reduced roGFP [37]. Using this tool, it has been found that PEXO-derived ROS can disturb MITO redox balance, and it will be interesting to determine whether and how PEXO-derived ROS influence total and MITO redox balance in the muscle cells.

It is intriguing to find increased Catalase mRNA and protein levels but at the same time, a surge in the ROS level in myotubes. The detection of myotube-specific Catalase-b (Figure 2E) suggests the existence of a large amount of nonfunctional Catalase, which might be either the cause or effect of a high ROS level in myotubes. Treating myotubes with antioxidants, such as vitamin C/E, will probably reveal whether it is induced by high ROS generated by other mechanisms. Currently, it is unknown how Catalase-b is derived and neither its sequence is known. Although splicing variants of Catalase gene are not commonly found in wildtype subjects, mutation of the CDS or splicing junctions do create nonsense/missense mutations of *Catalase,* which causes a disease called acatalasia/acatalasemia in humans [38]. As this lower molecular weight Catalase-b has not been reported before, it will be interesting to see whether its over-expression can cause PEXO functional defects and a subsequent ROS surge in muscle and other cells.

## 4. Methods

### 4.1. Plasmids

The promoter of *Pex3* was amplified from mouse genomic DNA by *Taq* DNA polymerase and inserted into the *Xho*I site of the pStable-luc vector. The promoters of *Pex16* and *Pex19* were amplified from mouse genomic DNA by *Taq* DNA polymerase (*pfu*) and inserted into the *Sma*I site of the pGL3-basic vector and the *Xho*I (blunted) site of the pStable-luc vector, respectively. The expression vector (TRCN0000126812) for *shRNA* targeting *Pex3* was acquired from the RNAi core facility of the Academia Sinica (https://rnai.genmed.sinica.edu.tw/index.html (accessed on 5 August 2018)). The coding sequence of RFP was PCR amplified from PLKO-*RFP* vector with primers containing the PTS1 sequence in the N-terminus and inserted into the *EcoR*V site of the pCDNA3.0 vector [26]. The coding sequence of the Flag-HA-mEeos4b was amplified from parental vector pCDNA3-Flag-HA-mEeos4b (a general gift from Dr. Won-Jin Wang, National Yang Ming University) by *Taq* DNA polymerase (*pfu*) with PTS1-Tag encoded in the reverse primer. The PCR product (Flag-HA-mEeos4b-PTS1) was inserted into the *EcoR*V site of the pCDNA3 vector. The CDS of *Pex3* was amplified from the p*PEX3*–GFP (a generous gift from Dr. Jennifer Lippincott-Schwartz, Janelia Research Campus, Howard Hughes Medical Institute) by *Taq* DNA polymerase (*pfu*) and inserted into the *Xho*I (blunted) site of the pCEGFP-TRE vector to generate the pCEGFP-TRE-*Pex3* plasmid. Both pCEGFP-TRE and pStable-luc vectors were created in our laboratory and their description can be found in our previous studies [39]. The primer sets used in the cloning are listed in the Supplementary Table S2.

### 4.2. Cell Culture and Differentiation

C2C12 myoblasts and C3H10T1/2 fibroblasts were kept in growth medium (GM) containing DMEM supplemented with 20% and 10% FCS, respectively. C2C12 were kept at low confluence during passage to avoid spontaneous differentiation and cells at this stage were called proliferating myoblasts (PMB). To induce myogenic differentiation, C2C12 were allowed to grow confluent to become confluent myoblasts (CMB), then GM was replaced by differentiation medium (DM, DMEM supplemented with 2% horse serum) to trigger the formation of multinucleated myotubes (MT) for 3–4 days before it was harvested for various analyses.

### 4.3. Satellite Cells Isolation and Culture

Satellite cells were isolated with the pre-plating protocol as reported previously [40]. Briefly, neonatal FVB mice were euthanized with a CO_2_ overdose and their SKM was isolated and cut into small pieces before digestion with collagenase (2 mg/mL) and trypsin (0.5×) at 37 °C for 2 h. Suspended cells and tissues were collected via centrifugation and further digested with new enzymes for 2 h at 37 °C until tissue clumps disappeared. An equal volume of plating medium (DMEM with 10% horse serum) was added and the mixture was filtered through cell strainers to remove undigested tissues. Cells in the filtrate were collected via spinning and re-suspended in growth medium (DMEM with 20% FCS and 3% chicken embryo extract) before being seeded onto dishes for attachment of adherent cells (named pre-plating #1, PP1). After 1 h, suspended cells were transferred to new dishes (PP2) and incubated for 2 h before being transferred to new dishes (PP3) for further incubation for 24 h. Then, cells were transferred to new dishes coated with collagen I/II (PP4) and incubated for 24 h before being transferred again to collagen-coated dishes with collected PP5 cells. Both PP4 and PP5 cells contained major satellite cells (>95%) and were further expended and used as satellite cells in various assays. The use of mice for isolating satellite cells and embryos has been approved by the Institutional Animal Care and Use Committee of National Central University with the approval numbers NCU-108–015 and NCU-109–012. Satellite cells were kept at low confluence to avoid spontaneous differentiation and their myogenic differentiation was induced when they were in high density, but not confluent, by changing to differentiation medium as seen in C2C12 myoblasts.

### 4.4. Transfection and Transient Promoter Assay

Cells were split and plated into 12-well culture dishes one day before transfection, so all transfections were performed at 60–70% of cell confluence. DNA aliquots (0.67 µg reporter construct and 0.33 µg expression vector per well) and transfection reagent (T-Pro NTR-II, T-Pro Biotechnology) were mixed in 1X Hepes buffer (20 mM Hepes at pH 7.0, 187 mM NaCl, 5 mM KCl, 0.7 mM Na_2_HPO_4_, and 5.5 mM dextrose) and incubated at room temperature for 15–30 min, allowing the DNA/liposome complex to form. Then, aliquots of culture medium were added to each tube and mixed gently, and medium containing the DNA/liposome complex was transferred to cells in triplicate. This transfection step was allowed to proceed overnight before the media were replaced with differentiation medium containing 2% horse serum. Cells were harvested and assayed for luciferase activity 16–24 h after transfection in a Clarity 2 luminometer (BioTEK; Winooski, VM, USA).

### 4.5. Stable Transfection of Over-Expressed Cells

C2C12 myoblasts stably over-expressed with *RFP-PTS1* (C2-RFP-PTS1) was created by transfecting pCDNA3-RFP-PTS1 into C2C12 at low confluence, as described in the transfection before selection with G418 (800 μg/mL) for 2–3 weeks until multiple stable clones were acquired. Monoclonal colonies were picked and expended to identify clones with high RFP-PTS1 expression and normal myogenic differentiation. C2C12 myoblasts stably over-expressed with *Flag-HA-mEeos4b-PTS1* (C2-Flag-HA-mEeos4b-PTS1) were created similarly.

To establish the *Tet-off* stable clones, the tetracycline-controlled transactivator (*tTA*) expression vector pEF-tTA-IRES-puro (5 μg/well) was transfected into C2C12 myoblasts and selected with puromycin (2.5 μg/mL) for 2–3 weeks to generate multiple colonies. Monoclones (C2-*tTA*) with stable *tTA* expression and normal myogenesis were selected for further experiments. To generate stable clones with *Pex3* expression under *tTA* regulation, pCEGFP-TRE-Pex3 vector (5 μg/well) was transfected into the C2-*tTA* cells and selected with G418 (800 μg/mL) and puromycin (2.5 μg/mL) for 2–3 weeks until stable clones were acquired. Monoclones (C2-*tTA-Pex3*) with stable GFP and *Pex3* expression and normal myogenesis were selected for further analyses. Doxycycline (Dox, 25 ng/mL) was included in the medium to avoid the expression of *Pex3* and *GFP* during selection and passage, and their expression was allowed when Dox was removed.

### 4.6. Gene Knockdown by Lentivirus Expressed shRNA

Infectious lentivirus expressing *shRNA* targeting *Pex3* mRNA was generated by transfecting pCMV-Δ8.91 (5.625 μg), pMD.G (0.625 μg), and the *shRNA* expressing pLKO1 vectors (6.25 μg) into 293FT cells (on a 10 cm dish) for 48 hr before they were collected in the culture medium to infect C2C12 myoblasts. Infected C2C12 myoblasts were stored with puromycin (2 ug/mL) for at least 2 weeks to generate monoclonal colonies and these clones were expanded and examined for their *Pex3* levels via RT-PCR. Clones derived from pLKO1-126812 plasmid (clone ID TRCN0000126812) showing reduced *Pex3* levels were used for further experiments. All vectors used for the knockdown experiment were acquired from the RNAi core facility of Academia Sinica.

### 4.7. Knockdown of Pex3 by CRISPR Interference (CRISPRi)

The CRISPRi system was established according to previous studies [41], and the detailed protocol has been published in our previous study [42]. Briefly, C2C12 myoblasts were over-expressed with *KRAB-dCas9-P2A-mCherry* fusion gene via lentivirus infection and infected C2C12 myoblasts were later serially diluted in a 96-well titer plate to identify red clones expressing KRAB-dCas9-P2A-mCherry. Cells with bright mCherry fluorescence were further expanded and then transfected with the PX459ΔCas9 vector (a derivative of the PX459 vector with the *Cas9* gene deleted) carrying *sgRNA* (*sgPex3-1*: 5′-TCCGTCCGCAAATAGCTCCC-3′ or *sgPex3-2*: 5′-AGCTATTTGCGGACGGACCC-3′) targeting the sequence (−14~+6 and −11~+9, respectively) around the transcriptional initiation site of the *Pex3* gene and stored with puromycin (2.5 μg/mL) for 2–3 weeks. Monoclones with bright mCherry fluorescence and puromycin resistance were picked up and expanded to analyze the expression of *Pex3* and other genes via qRT-PCR. The targeting site and sgRNA sequences were designed using the CHOPCHOP (https://chopchop.cbu.uib.no (accessed on 10 January 2021)) and CRISPO (http://crispor.tefor.net (accessed on 11 May 2022)) algorithms.

### 4.8. Quantitative RT-PCR (qRT-PCR)

Total RNA was isolated by lysing cells with Solution D (4 M guanidinium thiocyanate, 25 mM sodium citrate, pH7.0, 0.5% sarcosyl, 0.1% β-mecaptoethanol) and the genomic DNA and proteins were removed via repeated (×4) acid phenol/chloroform extractions before they were cleaned up via ethanol precipitation and high speed centrifugation [43]. The detailed protocol for traditional and quantitative RT-PCR has been described in our previous studies [27,44]. Briefly, the first strand of cDNA was synthesized using the Superscript III kit (Invitrogen) according to the manufacturer’s protocol. All qPCR was performed in a 15 μL reaction mixture containing 5 μM forward/reverse primers, template cDNA, and 1X SYBR Green reaction mix (RealQ Plus 2× Master Mix Green, Ampliqon) to detect PCR product. The signal of the *m36b4* gene, encoding for a ribosomal protein, was used as internal control amplified in the same PCR assay. The expression level of each gene was normalized to that of *m36b4* first, then, the normalized expression level (△C_t_) in control cells was arbitrarily set as 1, and the control cell △C_t_ was deduced from those of the treatment groups to derived △△C_t_. The relative expression level of each gene in treatment group cells was calculated using the equation (2^−△△Ct^). To ensure the linearity of PCR amplification, 2 doses of template cDNA (equivalent to about 7 and 14 ng of total RNA) were used in separate tubes to obtain 1 cycle difference in their Ct value but with the same △C_t_. All reactions were performed in the Rotor-Gene Q (QIAGEN) real-time PCR machine. The primer sets used in qRT-PCR are listed in Supplementary Table S1.

### 4.9. Determination of ROS Level

The detailed protocols for these assays have been described in our previous study [25]. Briefly, cells were grown in 6-well plates until confluent (CMB) or induced to become myotubes (MT) before they were used for assays. To measure ROS, cells were incubated in KRPH buffer (20 mM Hepes at pH 7.4, 5 mM KH_2_PO_4_, 1 mM MgSO_4_, 1 mM CaCl_2_, 136 mM NaCl, 4.7 mM KCl, and 1% BSA) containing 30 μM H2-DCFDA for 30 min. Then, cells were extensively washed with PBS, trypsinized (with 0.25× trypsin) and re-suspended in PBS buffer. The fluorescence of H2-DCFDA in the cells was measured using a fluorescence spectrophotometer (Hitachi F-4500) and normalized based on the protein level of lysate after cells were lysed post ROS determination.

### 4.10. Fatty Acid β-Oxidation

The protocol for measuring fatty acid oxidation has been described in our previous studies [25,26]. Briefly, BSA-conjugated 2 μCi ^3^H-labeled oleic acid (NET289005MC, Perkin Elmer) or palmitic acid (NET043001MC, Perkin Elmer) was added into cells kept in αMEM and incubated for 6 h in the presence of cold oleic (100 μM) or palmitic (50 μM) acid, respectively. Then, medium was collected and purified with 2 steps of methanol/chloroform ((3:2) followed by (1:1)) extraction to remove un-metabolized ^3^H-palmitic acid or -oleic acid, as reported before [45]. The ^3^H radioactivity in the aqueous phase was determined using a scintillation β-counter (LS6500, Beckman) and normalized with cell lysate protein concentration to represent fatty acid β-oxidation activity of each treatment.

### 4.11. Redox Ratio Assay

This assay was performed as previously described [46]. Briefly, cells grown on 6-well plates were harvested via trypsinization and suspended in PBS. Cell numbers were counted using a hemocytometer and their emission of 460 nm light after they were excited with 340 nm light was determined using the F-4500 fluorescence spectrophotometer (Hitachi) to measure their NAD(P)H content. Similarly, their emission of 528 nm light after they were excited with 454 nm light was detected to determine their FAD content. After normalization with the protein level of each sample, the redox ratio was calculated based on the emission intensity of FAD and NAD(P)H using the equation FAD/(FAD + NAD(P)H).

### 4.12. Mitochondrial Staining and DNA Quantification

Myoblasts grown on 6-well plates at various myogenic stages were washed with PBS thoroughly before incubated with KRPH buffer containing either MitoTracker Red CMXRox (50 ng/mL, Thermo Fisher Scientific, Waltham, MA, USA) or JC-1 (2 μM; #1130-5, BioVision, Waltham, MA, USA) for 30 min. After extensive wash in cold PBS, cells images were taken under a Carl Zeiss Axio Observer A1 fluorescence microscope with AxioVision 4.8 software. Then, cells were trypsined and re-suspended in PBS. Cell numbers were counted on hemocytometer and their emission of 595 nm light after excited with 575 nm light was determined using the F-4500 fluorescence spectrophotometer (Hitachi) to measure the MitoTracker Red staining level. The intensity of JC-1 staining of cells was measured by their emission of light at 590 (aggregates, red) and 530 (monomers, green) nm wavelengths after they were excited with light of 488 nm wavelength. The fluorescence intensity was normalized based on the protein concentration after cells were lysed. MITO morphology was also viewed through Mitotracker staining. Cells with all (or most) MITO shown as filament or vesicle in the cytoplasm were classified as filament or vesicle, respectively. While cells with both filament and vesicle MITO mixed in the cytoplasm were classified as mixture. If a cell with more than 4~5 vesicle MITO co-existed with filament MITO (or vice versa), it was classified as a mixture.

Total genomic DNA (nuclear and mitochondrial) was isolated from cells grown on 6-well plates at various myogenic stages. The relative amounts of mitochondrial DNA in the cells were determined via quantitative PCR detecting mitochondrial genes (*cytochrome b* and *Cox II*) and nuclear genes (*MyoD* and *Oct4*) levels in total genomic DNA of the same sample. The nuclear gene levels served as an input control to normalize levels of MITO genes to calculate the relative amount of MITO DNA in each sample.

### 4.13. Catalase Activity Assay

The assay was modified from previous studies [47]. Briefly, C2C12 cells were harvested via trypsinization and re-suspended in PBS. Then, cells were lysed via sonication and then centrifuged to remove cell debris. The protein concentration in the supernatant was determined via Bradford protein assay and 10 ug total lysate was included in the 150 uL PBS-based reaction mixture (100 mM H_2_O_2_ with or without 20 mM catalase inhibitor 3-amino-1,2,4-triazole (3AT)). At various times (5, 10, or 20 min) after the reaction, residual H_2_O_2_ levels were determined by reading the light intensity in a Clarity 2 luminometer (BioTEK; Winooski, VM) after injecting detection mixture (100 µL) containing HRP-conjugated IgG (1 × 10^4^-fold dilution in PBS) and enhanced chemiluminescence (ECL) reagent (Amersham Pharmacia Biotech). For reactions containing 3-amino-1,2,4-triazole (3AT), lysate and 3AT were pre-incubated for 20 min to ensure the inhibition of Catalase by 3-AT.

### 4.14. Succinate Dehydrogenase (SDH) Assay

This assay was performed as previously described [48]. Briefly, cells at various myogenic stages were harvested via trypsinization or lifting in PBS containing 2 mM EDTA and washed extensively with PBS before being sonicated to lyse the cells. After centrifugation, the protein concentration in the supernatant was determined via Bradford assay. For SDH activity assay, lysate (50 μg), sodium succinate (200 mM, pH > 7), sodium azide (8 mM), and 2,6-dinitrophenolindophenol (DCPIP, 0.004%) were incubated at 37 °C for 30~60 min before SDS (final 2%) was added to stop the reaction. The reaction product was determined by reading the optic density (O.D.) at 600 nm in an ELISA reader and the SDH activity was calculated using equation O.D. (blank-sample)/O.D. (blank).

### 4.15. Western Blot

The Western blot protocol has been described in detail in our previous study [39]. Briefly, aliquots of total lysate (50 μg) in RIPA buffer (with protease and phosphatase inhibitors) were run on 10% SDS-PAGE gels before being transferred onto a PVDF membrane (Pall FluoroTrans W membrane, PALL). After being extensively washed with PBST (PBS containing 0.5% Tween 20), PVDF membranes were blocked with 5% skimmed milk or 1% BSA (for phosphorylated proteins only) in PBST for 30~60 min. Primary antibody was diluted 1:1000 in blocking solution and incubated with the blot at 4 °C overnight. After several washes with PBST, HRP-conjugated secondary antibody (1:10,000 dilution) was added and incubated at room temperature for 1 h. After extensive washing in PBST, the HRP signal was detected using an enhanced chemiluminescence kit (Western Bright ECL, Advansta). Catalase antibody was obtained from Santa Cruz (H-9, SC271803) and the Flag antibody was purchased from Sigma (M2 antibody, F3165). The antibodies for SDHB2, Gapdh, Lamin B1, OXPHOS subunits, MTCO1, and MTCO2 were from Abcam (ab178423, ab9482-200, ab16043-25, ab110413, ab1475, and ab198286, respectively).

### 4.16. Detection and Counting of Peroxisomes

C2C12 myoblasts stably over-expressed with *RFP-PTS1* (C2-RFP-PTS1) or *Flag-HA-mEos4b* (C2-tTA-mEos4b) were grown on glass cover slides held in 12-well dishes to reach various myogenic stages. Then, cells were washed extensively with cold PBS before being fixed in 4% paraformaldehyde for 30 min. Afterward, cells were extensively washed with PBS before being quenched with NH4Cl (50 mM) in PBS. To visualize the nuclei, cells were incubated with DAPI (2 μg/mL in PBS) at room temperature for 10 min and were washed with PBS thoroughly before being mounted on a slide and sealed with nail polish. To induce a color change of mEos4b, cells were exposed to UV light for a short time and harvested 24 h later or as indicated in the figure legends. All the images were observed and photographed under a Carl Zeiss Axio Observer A1 fluorescence microscope using AxioVision 4.8 software (Carl Zeiss; Oberkochen, Germany). The peroxisome numbers on photographed images were counted either manually or automatically using the Image J software.

### 4.17. Immunofluorescence (IF) Staining

For IF staining of myosin heavy chain (MHC), cells differentiated in DM for 72~96 h were washed with cold PBS before being fixed in 4% paraformaldehyde for 20 min. Then, they were quenched in 50 mM NH4Cl for 15 min before being permeabilized in 2% Triton-X 100 overnight. Cells were incubated in blocking solution (2% BSA and 2% goat serum in PBS) for 20 min, followed by incubation with MHC antibody (1:1000 dilution; clone MY-32, Sigma) overnight. After extensive washing with PBS, Alexa488- or 546/548-conjugated secondary antibody (Invitrogen) was added and incubated for one hour before it was washed in PBS and mounted on a slide as described above. The signals of MHC were observed and photographed under a Carl Zeiss Axio Observer A1 fluorescence microscope with AxioVision 4.8 software. To visualize PEXO, PMP70 antibody (ab3421, abcam) was used and cells were processed as described above. Goat anti-rabbit Alexa^®^488 and goat anti-mouse Alexa^®^568 were both purchased from Invitrogen (A-11001 and A-11004, respectively).

### 4.18. The Isolation of Embryos and Satellite Cells

Satellite cells were isolated according to the pre-plating protocol, as reported before [40]. Briefly, neonatal FVB mice were euthanized with a CO_2_ overdose and their SKM was isolated and cut into small pieces before being digested with collagenase (2 mg/mL) and trypsin (0.5×) at 37 °C for 2 h. Suspended cells and tissues were collected via centrifugation and further digested with new enzymes for 2 h at 37 °C until tissue clumps disappeared. An equal volume of plating medium (DMEM with 10% horse serum) was added and the mixture was filtered through cell strainers to remove undigested tissues. Cells in the filtrate were collected via spinning and re-suspended in growth medium (DMEM with 20% FCS and 3% chicken embryo extract) before being seeded onto dishes for attachment of adherent cells (named pre-plating #1, PP1). After 1 h, suspended cells were transferred to new dishes (PP2) and incubated for 2 h before being transferred to new dishes (PP3) for further incubation for 24 h. Then, cells were transferred to new dishes coated with collagen I/II (PP4) and incubated for 24 h before being transferred again to collagen-coated dishes to collect PP5 cells. Both PP4 and PP5 cells contained major satellite cells (>95%) and were further expended and used as satellite cells in various assays. Embryos were isolated on 11.5 dpc from CO_2_-euthanized female mice and the day of females carrying a mating plug was counted as 0.5 dpc. After isolation, embryos were dissected under microscope into head, rostral, interlimb, and tail regions, and the same regions from different embryos were pooled to isolate total RNA. The use of mice for isolating satellite cells and embryos has been approved by the Institutional Animal Care and Use Committee of National Central University with the approval numbers NCU-108-015 and NCU-109-012. All animal experiments were conducted at the animal facility of the Department of Life Sciences, National Central University. The dissection and isolation of cells and embryos was performed at the Cell Differentiation Laboratory in the same department.

### 4.19. Statistical Analyses

All experiments were performed at least 3 times, with similar results. The results from independent experiments were pooled and their means and standard errors are shown. The difference between various treatments was examined using Student’s *t*-test. All tests were performed in one-to-one (treatment vs. control), but not paired, comparison and a probability value of <0.05 was considered statistically significant. 

## Data Availability

All supporting data are included within the main article and its supplementary files.

## Supplementary Materials

The following supporting information can be downloaded at: https://www.mdpi.com/article/10.3390/ijms241512262/s1.
