# Supplementary material for: Reciprocal Regulation of Peroxisome Biogenesis and Myogenic Factors Is Critical for Myogenesis
Source: Int J Mol Sci. 2023 Jul 31;24(15):12262. doi: 10.3390/ijms241512262 (PMC10419124; doi:10.3390/ijms241512262)
Supplement: Supplementary file 1 [file ijms-24-12262-s001.zip › ijms-2497345-supplementary.pdf]

Supplementary Materials

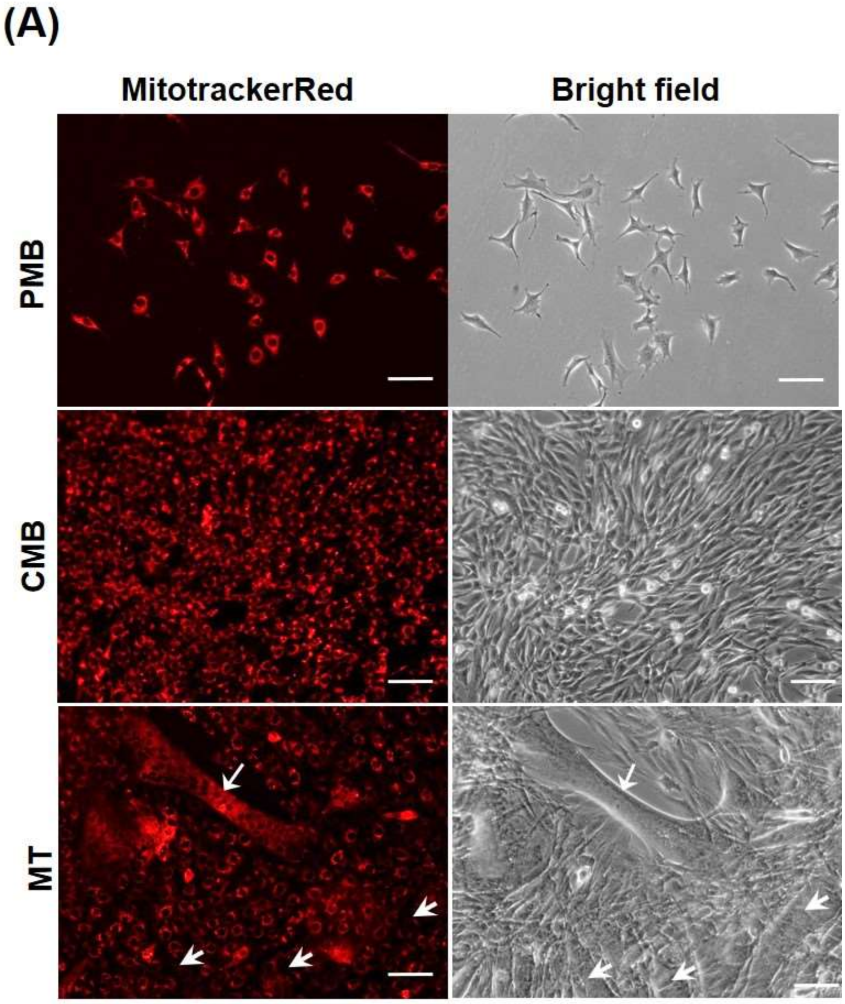

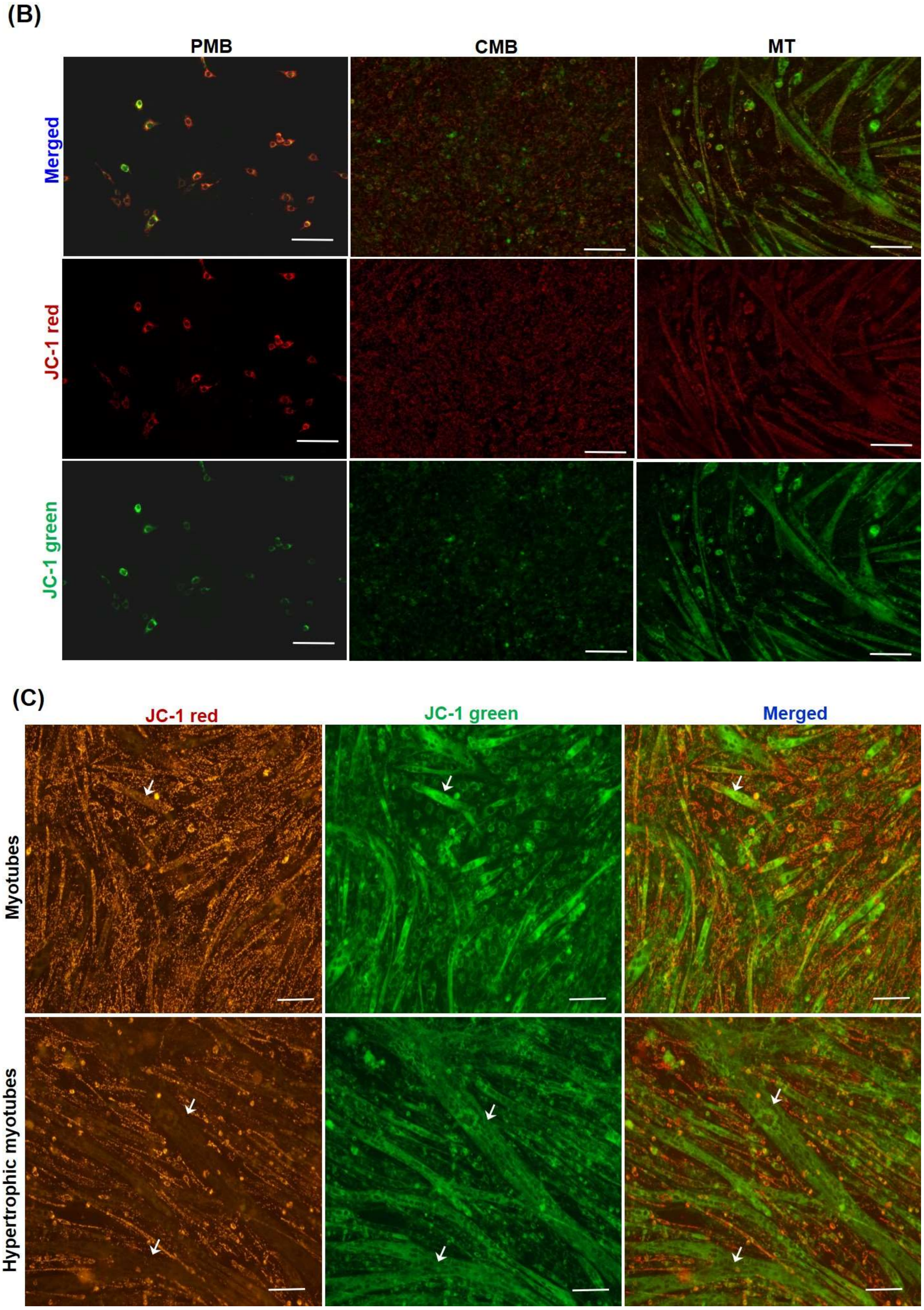

(B)
PMB
CMB
MT
Merged
JC-1 red
JC-1 green
(C)
JC-1 red
JC-1 green
Merged
Myotubes
Hypertrophic myotubes

(D)

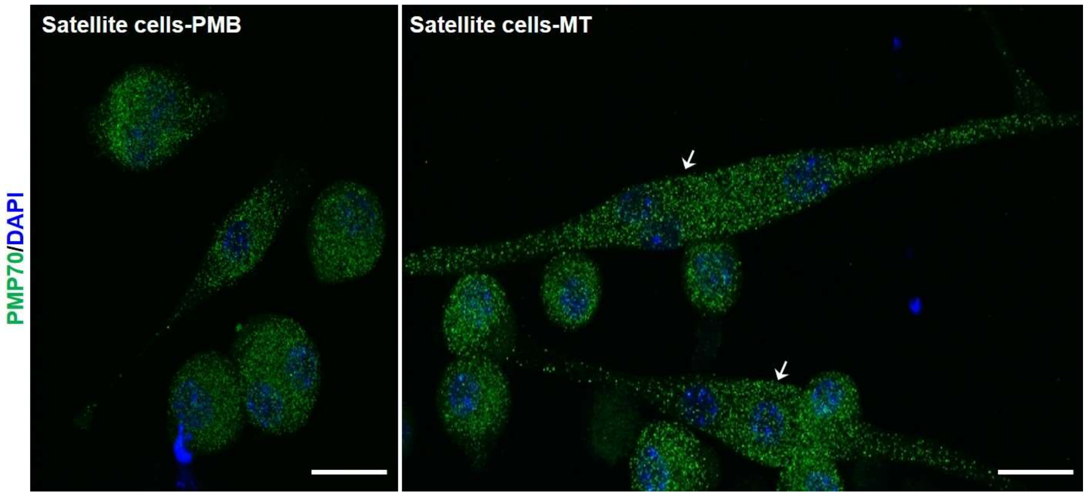

Supplementary Figure S1: The images of MITO and PEXO stained with fluorescent dyes. C2C12 myoblasts at proliferating myoblast (PMB), confluent myoblast (CMB), and differentiated myotube (MT) stages were treated with Mito Tracker-Red CMXRox (50 ng/ml, 30 min) or JC-1 (2 µM, 30 min) fluorescent dye and their MITO morphology is shown in **(A)** and **(B)**, respectively. **(C)** JC-1 stained myotubes (top panel) and hypertrophic myotubes (lower panel) were viewed and photographed under high resolution microscope and camera to see the JC-1 red aggregates. **(D)** Confocal microscope images of satellite cells. Scale bar: 100 µm in (A) ~ (C) and 20 µm in (D). Arrow: myotubes; arrow head: myotubes lacking Mito tracker staining.

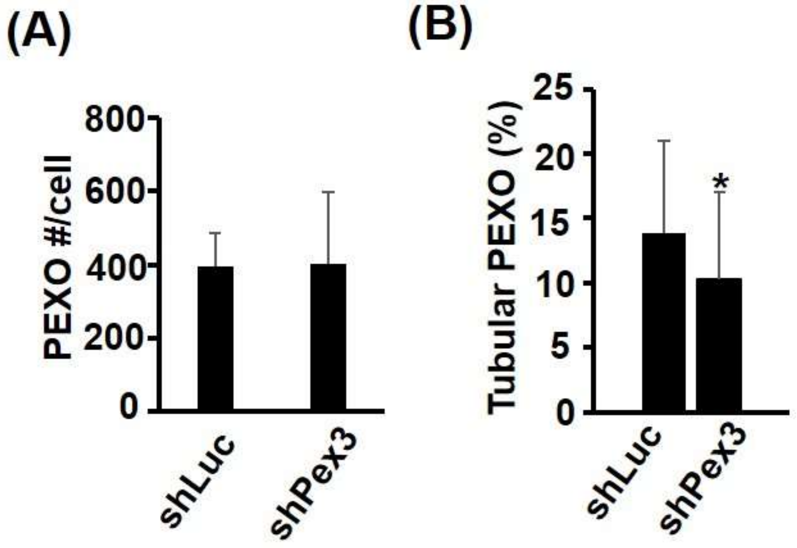

Supplementary Figure S2: The alteration of PEXO in C2-shPex3 cells

The PEXO number and ratio of tubular PEXO in *Pex3* knockdowned stable clones (C2-shPex3) are shown in **(A)** and **(B)**, respectively. n=20 for (A)~(B).

Supplementary Table S1: Primer sets used in the quantitative RT-PCR.

NCU Code	Gene Name	Primer Sequence[a]	Amplicon Size
000008<br>000009	*Mef2c*	FP: 5′- ccc atc aga ccg cct gtg tt -3′<br>RP: 5′- gct ttg aga tgc cag tta cc -3′	184bp
000012<br>000013	*Mrf4*	FP: 5′- agc agt ctt cag cgc ctt tct -3′<br>RP: 5′- atg ttc caa atg ctg gct gag t -3′	111bp
000053<br>000054	*COX1*	FP: 5′- cgc aat tcc tac cgg tga ca -3′<br>RP: 5′- tga agc acg atg tca agg ga -3′	168 bp
001056<br>001057	*COX2*	FP: 5′-aac cga gtc gtt ctg cca ata g-3′<br>RP: 5′-acc ctg gtc ggt ttg atg tta c-3	154 bp
003024<br>003025	*MyoD*	FP: 5′- ggc tac gac acc gcc tac ta -3′<br>RP: 5′- gtt ctg tgt cgc tta ggg at -3′	204 bp
006003<br>006004	*MyHCIIB*	FP: 5′- gcc gag caa gag cta ctg gat -3′<br>RP: 5′- tgt tga tga ggc tgg tgt tct g -3′	74 bp
006005<br>006006	*MyHCXII*	FP: 5′- tgc caa ggg cct gaa tga -3′<br>RP: 5′- gct tcc acc taa agg gct gtt -3′	104 bp
006012<br>006013	*Myogenin*	FP: 5′- cca gtg aat gca act ccc aca gc -3′<br>RP: 5′- aga cat atc ctc cac cgt ga -3′	167 bp
014023<br>014024	*m36B4*	FP: 5′- gaa cgt ggg ctt cgt gtt ca -3′<br>RP: 5′- ctc gac aca gcc gca ctc ttc -3′	167 bp
015052<br>015053	*Myf5*	FP: 5′- tgc acc acc acc aac cct aa -3′<br>RP: 5′- cag ggc tgt tac att cag g -3′	196 bp
029086<br>029087	*Prdm16*	FP: 5′- gca tat cca cag cag gg -3′<br>RP: 5′- cga tgc ttg ttg agg g-3′	171 bp
029088<br>029089	*PGC-1α*	FP: 5′- cgt gac cac tga caa cga g -3′<br>RP: 5′- ctg cat ggt tct gag tg-3′	164 bp
029116<br>029117	*MCAD*	FP: 5′-agc tga ttg gca atg tct cc-3′<br>RP: 5′-gat cgc aat ggg tgc ttt tg-3′	291 bp
037021<br>037022	*Cat*	FP: 5′- aga tga agc agt gga agg ag -3′<br>RP: 5′- ctc tca gga atc cgc tct -3′	184 bp
041044<br>041045	*Pex11β*	FP: 5′- ctt tgc aga gac atg ggg cta gt -3′<br>RP: 5′- gag cta gcc cag act ttc cag cc -3′	243 bp
041050<br>041051	*Acox1*	FP: 5′- aca gtg ctg tga ggc gcc agt ct -3′<br>RP: 5′- aaa agc ctt cag ccc agc -3′	233 bp
041075<br>041076	*Pex19*	FP: 5′- cca gag tca cca gga ctc ca -3′<br>RP: 5′- ggc cca agg cct gta act -3′	179 bp
041081<br>041082	*Pex16*	FP: 5′- cct ccg cta cca gga gta tg -3′<br>RP: 5′- aca gcg aca cag gca act tt -3′	209 bp
041085<br>041086	*Pex3*	FP: 5′- tcg ctg ctc cgc tct agt cc -3′<br>RP: 5′- gca tct cca gac ccc gtg tt -3′	197 bp

063001<br>063002	*Cpt1b*	FP: 5′- gaa gag atc aag ccg gtc atg g -3′<br>RP: 5′- aag tct gtc tct ttg cct ggg a -3′	104 bp
063009<br>063010	*Ndufb8*	FP: 5′- gaa ctg ggg tga acc gat ac -3′<br>RP: 5′- gca gca cgt aga ggg aag ga -3	225 bp
064001<br>064002	*EGFP*	FP: 5′- caa gat ccg cca caa cat cg -3′<br>RP: 5′- gac tgg gtg ctc agg tag tg -3	120 bp
064003<br>064004	*MCK*	FP: 5′- aca tcg tcc aga gtg aag cc -3′<br>RP: 5′- cca cag cac aga cag aca ct -3′	224 bp
066017<br>066018	*Nkx2.5*	FP: 5′- gac aaa gcc gag acg gat gg -3′<br>RP: 5′- ctg tcg ctt gct tgc act tgt agc -3′	222 bp
071013<br>071014	*h/mPex3*	FP: 5′- gca gaa tac att gcc caa gc -3′<br>RP: 5′- ctg cat taa ggc ctc tct cag -3′	111 bp
071044<br>071045	*Gata4*	FP: 5′- aaa tct aag acg cca gca ggt cc -3′<br>RP: 5′- ctt gat ggg gcg cat ctc tt -3′	123 bp
033034<br>033035	*MyoD promoter (nuclear DNA)*	FP: 5′-cga cgc gtc ctc ctc tgt gct gcc tta-3′<br>RP: 5′-cga cgc gtc ttt cag gct gtg cac-3	395 bp
021045<br>000003	*Oct4 promoter (nuclear DNA)*	FP: 5′-cga cgc gtc gag ggt gca gtg cca aca g-3′<br>RP: 5′-gcg acg cgt ggg cac ccc gag ccg ggg g-3	348 bp
037029<br>037030	*Cytochrome b (MITO DNA)*	FP: 5′-tcc tat cag cca tcc ca-3′<br>RP: 5′-gtt tga tcc tgt ttc gtg g-3	173 bp
001056<br>001057	*COX2 (MITO DNA)*	FP: 5′-aac cga gtc gtt ctg cca ata g-3′<br>RP: 5′-acc ctg gtc ggt ttg atg tta c-3	154 bp

[a]FP: forward primer; RP: reverse primer

Supplementary Table S2: Primer sets used in Cloning.

NCU Code	Gene Name	Primer Sequence[a]	Amplicon Size
062007<br>062008	*Pex3 promoter*	FP: 5′- ccg ctc gag cgg tgt acc tct ccc aac aga cct -3′<br>RP: 5′- ccg ctc gag cgg cgc tta ctg atc cgg gac -3′	2692 bp
071001<br>071002	*Pex16 promoter*	FP: 5′- ggt cct gtg ccc act acc t -3′<br>RP: 5′- ctt ctc tct ccg ccc tga a -3′	4092 bp
071003<br>071004	*Pex19 promoter*	FP: 5′- gtc tta gct tct gct ccc att g -3′<br>RP: 5′- tct tac tgc ctc cga ctt gc -3′	4070 bp
071005<br>071006	*flag-HA-mEos4b-PTS1*	FP: 5′- atg gac tac aag gac gac gat g -3′<br>RP: 5′- tca cag ctt gga tcg tct ggc att gtc -3′	768 bp
071007<br>071008	*hPex3 CDS*	FP: 5′- atg ctg agg tct gta tgg aat -3′<br>RP: 5′- tca ttt ctc cag ttg ctg agg ggt ac -3′	1122bp
TRCN0000126812	*shPex3*	5′- ccg gag cag cag tat tta tca agt act cga gta ctt gat aaa tac tgc tgc ttt ttt g -3′	18bp

[a]FP: forward primer; RP: reverse primer
